# Supplementary material for: Adaptive morphing of wing and tail for stable, resilient, and energy-efficient flight of avian-inspired drones
Source: Npj Robot. 2024 Nov 20;2(1):8. doi: 10.1038/s44182-024-00015-y (PMC11590198; doi:10.1038/s44182-024-00015-y)
Supplement: Supplementary file 1 — Supplementary Information [file 44182_2024_15_MOESM1_ESM.pdf]

Supplementary Information

Contents

1 Supplementary Notes: Quantification of disturbances 2
2 Supplementary Notes: Challenges of avian-inspired drone control 2

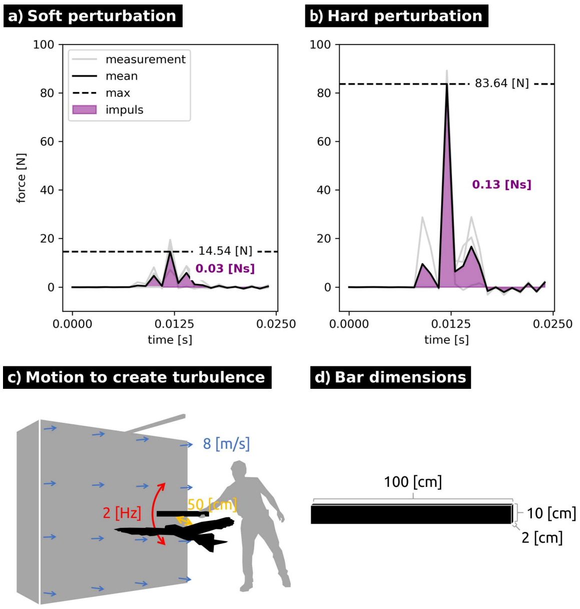

Supplementary Fig. 1: **External disturbances in steady-state flight.** Forces are measured during **(a)** *soft* and **(b)** *hard* physical perturbations. The signal (Newtons) from the force sensor was measured over a time window of 0.025 s (gray line). This experiment was repeated three times, the average (black line) was used to integrate over time (purple area), resulting in the applied impulse (Newton Seconds). **(c, d)** To disturb the airflow over the leading edge of right wing, a bar was waved at 2 Hz at a distance of 50 cm.

1 Supplementary Notes: Quantification of disturbances

In order to quantify the magnitude of the external perturbations applied to the drone, an ATI Nano25 force and torque sensor [1] was exposed to soft (Supplementary Fig. 1a) and hard (Supplementary Fig. 1b) physical perturbations similar to those exerted on the drone (Fig. 2). The measured force data were averaged over three trials and used to determine the impulse. The artificial turbulence in (Fig. 3) was created through waving a bar (Supplementary Fig. 1c,d), disturbing the airflow over the right wing leading edge.

2 Supplementary Notes: Challenges of avian-inspired drone control

The actuation of the morphing tail and wings, which have relatively large masses, introduces momentary torques due to the conservation of angular momentum, which is unrelated to aerodynamic effects. These inertia-based influences alter acceleration and velocity sensor readings, posing a challenge for control algorithms. To assess the inertia effects of the control surfaces, the drone was suspended from a cable at its centre of gravity. Each aerial surface (elevator, rudder, tail sweep, left and right wing sweep, allowing left and right wing twist) was actuated in a single step from the central position to the maximum range of motion, with no airflow present. We observe angular velocities surpassing 1 rad/s (Supplementary Fig. 2), suggesting that momentary inertia-based effects are stronger than the slower aerodynamic effects (Fig. 3). To address this issue, a first-order Butterworth low-pass filter (cutoff frequency 1.75 Hz) [2] is applied to the measurement data, filtering out momentary effects at the cost of introducing delay. The resulting trade-off yields a smoother, slightly delayed angular velocity signal that predominantly reflects lasting aerodynamic effects, thus enabling body-rate control. However, despite filtering, inertia-based

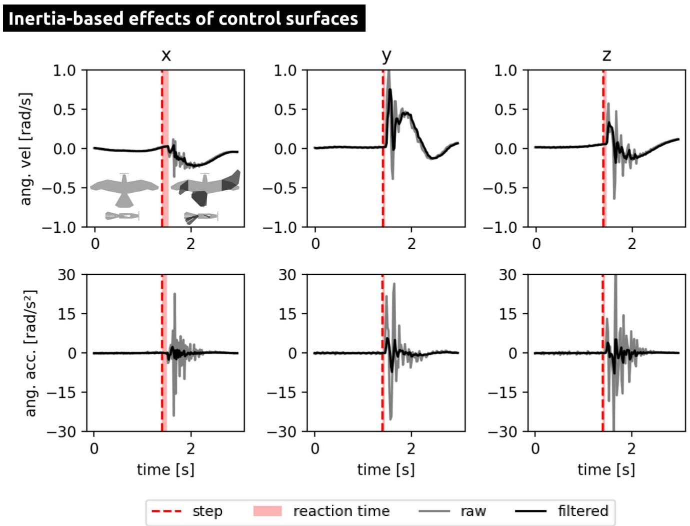

Supplementary Fig. 2: **Influence of actuator inertia on the angular velocity and acceleration.** Step-like movements (red dotted line) of all actuators (mean to maximum position) with no airflow present. Tail and wing sweep actuation induce momentary angular accelerations around the y- and z-axes. The resulting angular velocities in the x-axis direction are slower and a byproduct of the *LisEagle* rotation after the shift in the centre of gravity. The raw sensor measurements are passed through a low-pass filter.

effects remain dominant in the acceleration data, rendering acceleration-based controllers such as Incremental non-linear Dynamic Inversion (INDI) impractical without additional modelling refinement.

Modelling Avian-inspired drones (AIDs) is notoriously difficult. The quality of the proposed model is assessed by flying the *LisEagle* in steady-state flight (Supplementary Fig. 3a) and in oscillatory motion (Supplementary Fig. 3b) at a constant velocity of 8 m/s. The measured accelerations are compared against the model predictions. Constant discrepancies are observed due to asymmetries arising from maintaining and repairing the drone over countless flights, causing changes in the dynamics. Dynamic discrepancies are a result of unmodelled dynamic effects such as the movement of inertia-heavy actuators, aeroelasticity, and changes in airflow through the feathers. Nonetheless, our method is robust to these modelling discrepancies, as shown in the Results section.

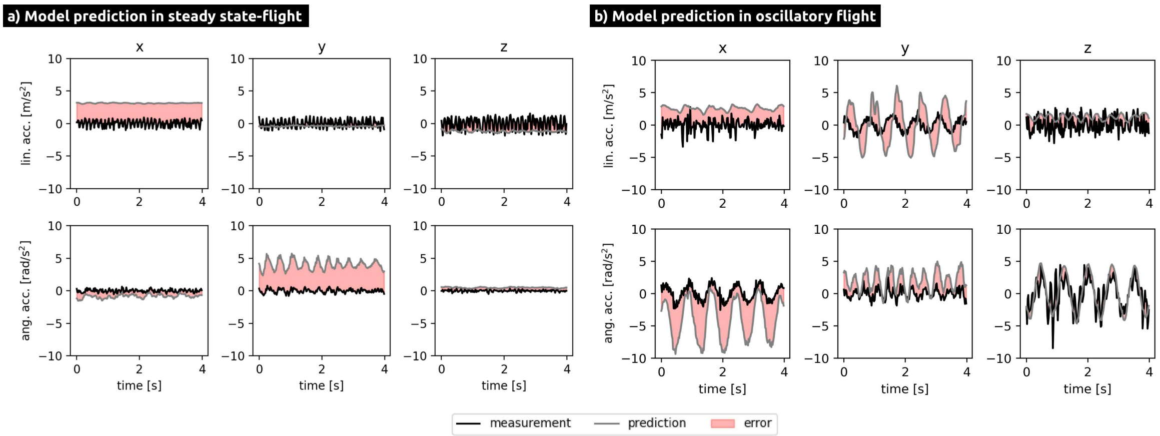

Supplementary Fig. 3: **Simulation to reality gap.** Discrepancy between model predictions and measured accelerations during **(a)** steady-state and **(b)** oscillating horizontal flight at 8 m/s. They are due to asymmetries arising from continuous maintenance, movement of inertia-heavy actuators, aeroelasticity and changes in airflow through the feathers.

References

[1] ATI (2024) Force and torque balance. URL https://www.ati-ia.com/products/ft/ft_models.aspx?id=Nano25, accessed: 2024-01-22

[2] Karki J (2000) Active low-pass filter design. Texas Instruments application report
